# Silent Breathlessness: A Case and Brief Review of Spontaneous Pneumomediastinum

**DOI:** 10.7759/cureus.4487

**Published:** 2019-04-17

**Authors:** Vincent Chan, Ahmad Raza, Bilal H Lashari, Rajesh Patel

**Affiliations:** 1 Internal Medicine, Abington Hospital - Jefferson Health, Abington, USA; 2 Pulmonary and Critical Care, Abington Hospital - Jefferson Health, Abington, USA

**Keywords:** spontaneous pneumomediastinum, respiratory medicine, respiratory physiology, pulmonary diseases

## Abstract

Spontaneous pneumomediastinum is an uncommon diagnosis defined as the presence of free air in the mediastinum without an apparent cause. It is a self-limiting disorder that most often occurs in young males without any apparent precipitating factor or underlying disease process. Its pathophysiology involves the rupture of alveoli with resultant air penetration into the mediastinum. Underlying disease processes, such as asthma, physical trauma, including yelling, contact sports, and Valsalva during labor, have also been reported to cause spontaneous pneumomediastinum. Here, we present the case of an 18-year-old male who presented to us with the chief complaint of cough and the subsequent diagnosis of spontaneous pneumomediastinum.

## Introduction

Spontaneous pneumomediastinum is a condition defined as the presence of free air in the mediastinum without a known cause. It is a relatively rare clinical entity found more prevalently in males as compared to females [1]. The disease is thought to be under-reported due to patients most commonly mistaking it for other disease processes, such as upper respiratory viral infections and asthma exacerbations, among other common diseases. The risk factors that have been associated with this disease process include contact sporting events, underlying pulmonary diseases, and, rarely, birthing [2-3]. Here, we describe a case of a young male who presented to our emergency department after being evaluated at an urgent care center for shortness of breath and cough.

## Case presentation

We present the case of an 18-year-old male high-school senior who presented to the emergency department (ED) for complaints of productive cough with associated dyspnea for 10 days. He was seen the day previous at an urgent care center where he received a chest X-ray (CXR) and was discharged with azithromycin for presumed pneumonia. He was given a call back the following day for referral to the ED because the CXR was read as pneumomediastinum.

**Figure 1:**
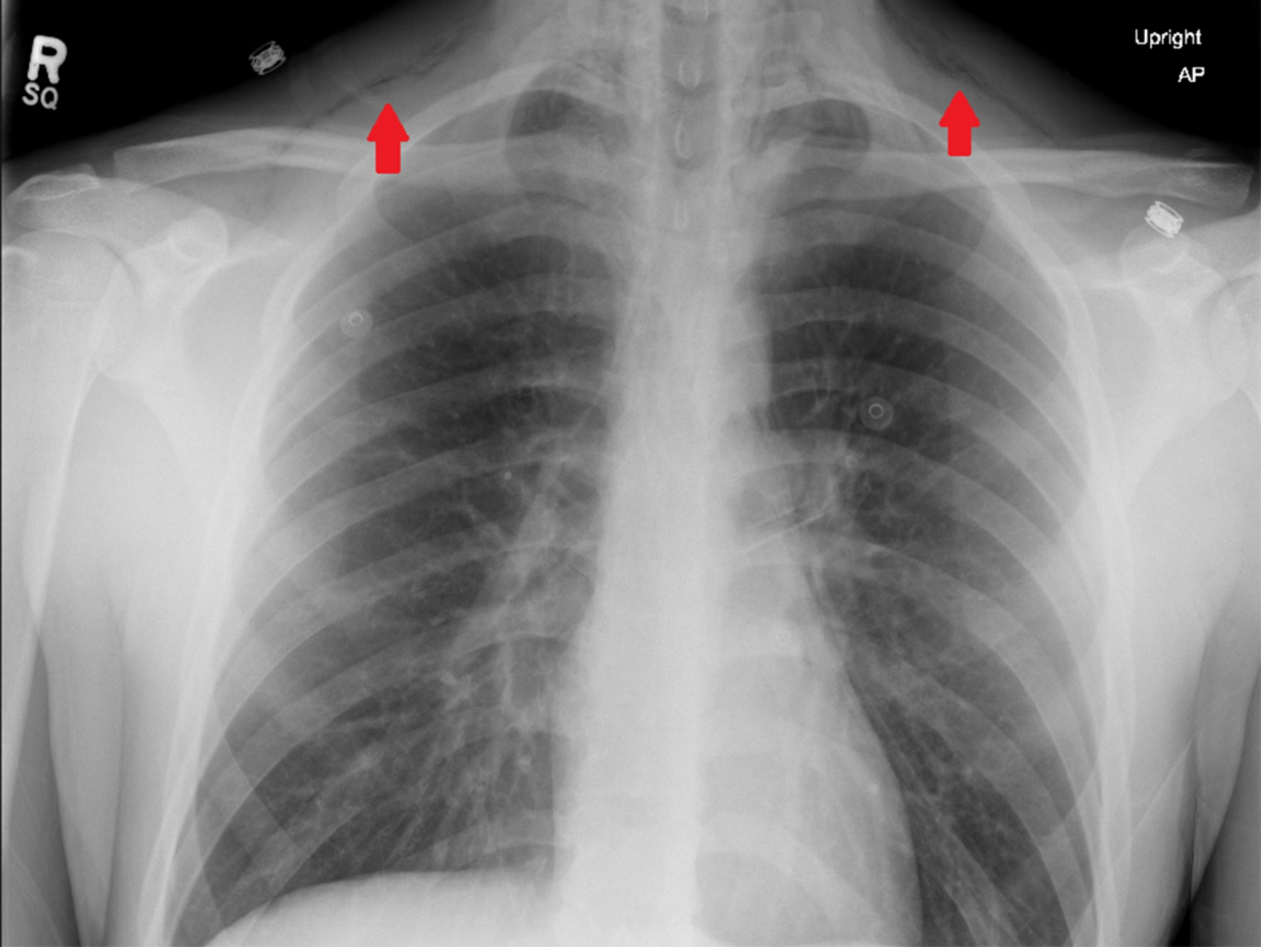
Upright anterior-posterior chest X-ray demonstrating air tracking in the subcutaneous tissue indicative of subcutaneous emphysema

**Figure 2:**
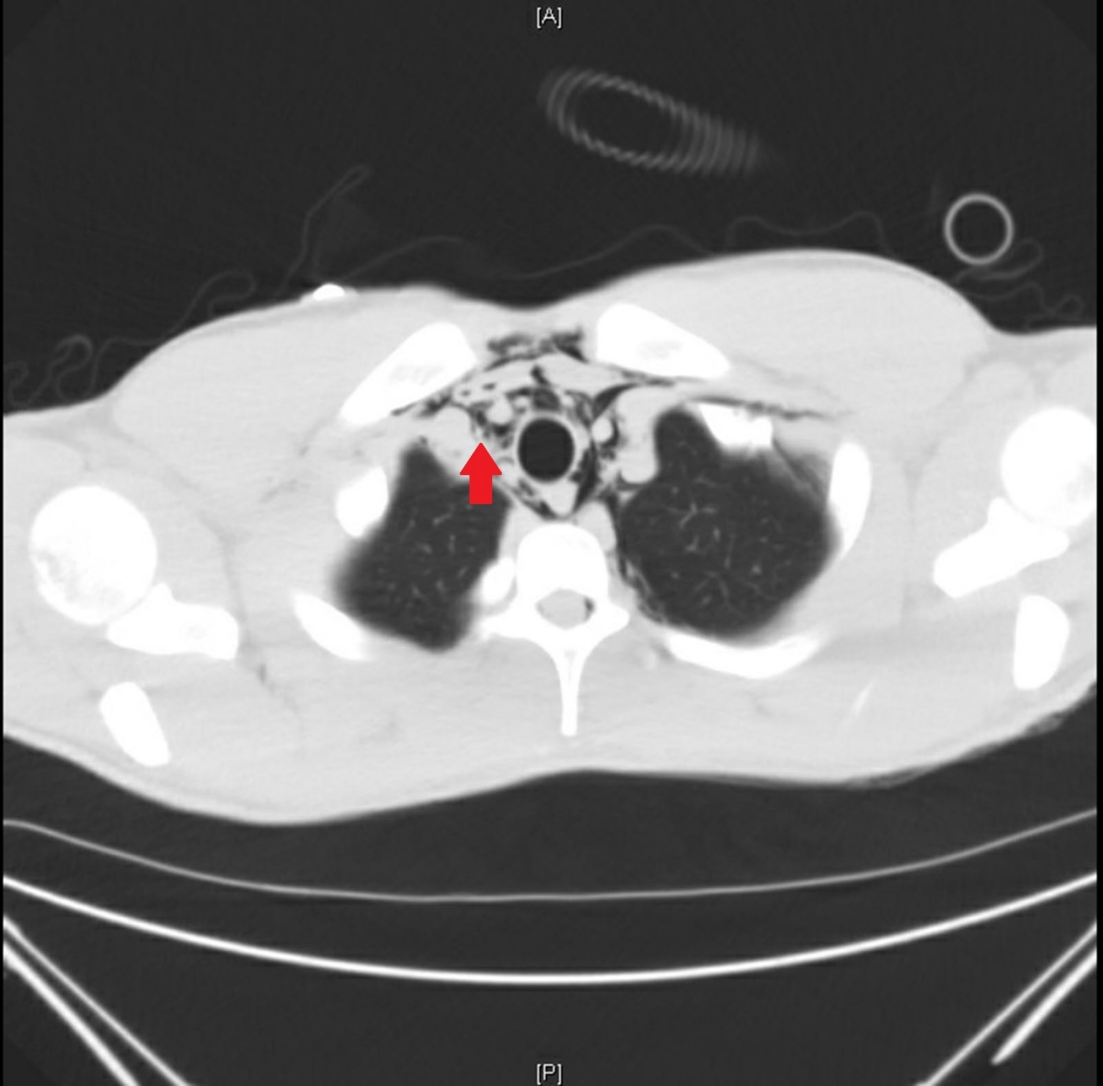
Computed tomography scan of our patient with spontaneous pneumomediastinum as demonstrated by free air surrounding the trachea anterior to lung parenchyma

In the ED, the patient’s triage vital signs included a blood pressure of 148/89, heart rate of 72 beats per minute, respiratory rate of 20 breaths per minute, oxygen saturation of 92% on room air, and a temperature of 99.6 Fahrenheit orally. The patient was an otherwise physically active and healthy male with a medical history of childhood epilepsy and seasonal allergic rhinitis. He noted that roughly 10 days ago, he began developing a dry cough, which progressed to include scant green sputum production. He had dyspnea, dysphagia, sore throat, intermittent wheezing, and positive sick contacts, especially through his participation in team sporting events. He participated in weight lifting, basketball, and lacrosse at a relatively high level. He noted that about three weeks ago, he took a “big hit” to the chest during a game of lacrosse but denied any symptomatology at that time. He did not associate his current symptoms to any traumatic event. Otherwise, his sporting regiment included significant physical exertion and vocal exertion but denied any extranormal periods of physical or vocal exertion outside of his typical regimen. No further history of recent physical traumatic events was noted. He denied any history of use of cigarette, cigar, marijuana, or illicit substances, including cocaine. He denied any complaints of headaches, dizziness, chest pain, palpitations at the time of evaluation. A CXR and computed tomography (CT) of the chest without contrast was obtained with findings of subcutaneous air in the thorax and around his neck (Figures 1-2). This confirmed that the patient had pneumo-mediastinum. On physical exam, he was 73 inches (185 cm) tall and weighed 175 lbs (79 kg). The patient was noted to have bilateral basilar minimal end expiratory wheezing without crackles or rhonchi. Palpable subcutaneous emphysema in the supraclavicular region and neck was present. There was no jugular venous distension, abnormal heart sounds, or lower extremity edema.

The patient was subsequently admitted for observation of dyspnea. His initial bloodwork was significant for leukocytosis with a white blood cell (WBC) count of 15 700 (normal, 3400 to 9600 cells/mcL). The patient was treated with 100% oxygen and albuterol-ipratropium nebulizer treatments and obtained re-imaging of his chest with a CXR. Re-imaging of his chest revealed stable air tracking through his mediastinum. The patient’s initial complaints of dysphagia had improved during his hospital stay and he was able to tolerate oral intake without any nausea or vomiting. The patient’s leukocytosis resolved within 24 hours and his symptomatology of cough and dyspnea had improved. The patient was subsequently discharged with outpatient pulmonology follow-up. The patient’s overall length of stay was 46 hours.

On outpatient follow-up, repeat CT of his chest four weeks thereafter demonstrated complete resolution of free air in the mediastinum. The patient had no other complaints. He was able to participate in a light exercise program and was to continue routine follow-up with his primary care physician.

## Discussion

Spontaneous pneumomediastinum (SPM), also known as Hamman’s syndrome, is a clinical condition defined as the presence of free air in the mediastinum with associated subcutaneous emphysema without an obvious precipitating cause. First described and published by Dr. Louis Hamman in 1939 [4], the pathophysiology behind SPM is thought to be secondary to the Macklin effect. This was described by Macklin et al. in 1944, who hypothesized that mediastinal air develops through a three-step process [5]: (1) blunt traumatic alveolar rupture, which results in an increase in intra-alveolar pressure; (2) the released alveolar air dissecting along the bronchovascular sheaths; and (3) the spread of alveolar air toward the pulmonary hila and into the mediastinum.

As imaging modalities improve, the incidence of spontaneous pneumomediastinum has increased, but it continues to be an infrequent entity, resulting in 1:800 to 1:42000 [6] hospital admissions and is specifically more often associated with males as compared to females [1]. The true incidence is likely not known, as spontaneous pneumomediastinum tends to be under-reported due to its generally benign course. Pneumomediastinum is generally described as a disease that usually occurs in young patients [2,7]. One explanation, as described in a previous review, is the fact that the mediastinal tissues are looser and flaccid in younger patients, allowing for the migration of air through these tissues. In the elderly, the planes and sheaths are fibrosed, making air migration more difficult [1]. In a study employing routine screening of young adults admitted for unexplained chest pain or dyspnea, the incidence of SPM was 1:368 [8]. Certain pre-disposing factors have been associated with the development of this condition, including asthma, interstitial lung disease, tobacco use, and inhaled drug use [2,9]. Physiological factors have also been reported in the literature, including emesis, exercise, cough, and labor [7].

Most commonly, it presents as retrosternal chest pain. This is reported to be present between 60% and 100% [2,10] of patients. Other frequent symptoms cited by case series include shortness of breath, neck pain, back pain, emesis, dysphagia, rhinolalia, and cough with variable frequencies. Physiologic signs, such as tachycardia and tachypnea, may also be observed. However, these symptoms may also be concurrently associated with a secondary disease process as its’ predominant cause, resulting in the attribution of symptoms to other diseases [2]. In patients with complaints of dysphagia, or an inability to tolerate oral intake, an esophagram is often used to exclude esophageal perforation but in a recent case series, the clinical utility was low in the setting of low clinical suspicion [11].

In cases of SPM, no complaint is usually reported and often no inciting factor is found. Clinicians should retain a high suspicion of spontaneous pneumomediastinum in cases of young males who present with acute chest pain [12] in the setting of a low index of suspicion of other life-threatening causes of chest pain. A common radiographic finding of subcutaneous emphysema, which is seen in up to 71% of patients, is the most common sign [2,13] and can help clinicians establish the diagnosis. Concurrent studies, such as routine laboratory evaluation and electrocardiogram, can help rule out other diseases. Periodically, SPM may also be associated with Hamman’s sign, an audible precordial crackle synchronous with heart sounds but not respiration. It is often best heard in the left lateral decubitus position. While specific, it is an uncommon entity, with its estimated prevalence ranging from 0.001% to 0.01% of cases [14].

Pneumomediastinum is thought to carry a good prognosis due to it being thought of as a benign entity in the vast majority of patients [15]. After the diagnostic workup has excluded significant pathology and other life-threatening illnesses, the treatment of SPM is aimed at the management of symptoms that patients may be experiencing as described [16]. Uncomplicated SPM is managed conservatively with analgesia, rest, and avoidance of maneuvers that increase pulmonary pressure (Valsalva or forced expiration, including spirometry) [17]. Asthma or any other underlying lung disease is treated as indicated when observed. At the time of discharge, patients are typically counseled to avoid Valsalva maneuvers and activities predisposing to barotrauma (eg, scuba diving). For patients with moderate to severe symptoms, therapy with high concentration oxygen has been used in an effort to enhance nitrogen washout [18]. However, if such patients have underlying chronic lung or airway disease that predisposes to atelectasis, 100% oxygen therapy should be administered with caution because it may lead to absorptive atelectasis. Complicated SPM is typically defined as SPM associated with tension pneumothorax, esophageal perforation, or pneumopericardium. Management, in that case, typically depends on the underlying etiology and the patient’s hemodynamic status.

In our patient, the cause of spontaneous pneumomediastinum was attributed to his regular participation in high-level sports activities, with likely above-average episodes of increased vocal exertion. No other preceding factor or causative agent was elicited from the patient. As such, he was managed conservatively with routine outpatient follow-ups to look for resolutions of both symptomatology and clinical findings. Though he did present with initial complaints of dysphagia, this improved during his hospital course without any further need for invasive imaging to entertain esophageal pathology. Repeat imaging completed four weeks after discharge revealed complete resolution of the free air previously in his mediastinum. He also had complete resolution of his symptomatology.

## Conclusions

Spontaneous pneumomediastinum is generally a benign entity where the focus should be to ensure an underlying disease process is adequately excluded or treated. While the disease is rare and likely under-reported, an invasive diagnostic workup is not needed in the setting of a low clinical suspicion of complications. Hamman’s sign is a physical exam finding that can intermittently help the clinician to suspect spontaneous pneumomediastinum, but the diagnosis is generally made via chest imaging. Prognosis is favorable in the absence of associated conditions, such as esophageal rupture, and is generally treated conservatively with oxygen with a resolution of symptoms.
